# The use of eculizumab in gemcitabine induced thrombotic microangiopathy

**DOI:** 10.1186/s12882-018-0812-x

**Published:** 2018-01-12

**Authors:** Vinod Krishnappa, Mohit Gupta, Haikoo Shah, Abhijit Das, Natthavat Tanphaichitr, Robert Novak, Rupesh Raina

**Affiliations:** 1 Department of Internal Medicine and Nephrology, Cleveland Clinic Akron General, 1 Akron General Ave, Akron, OH 44307 USA; 2 Department of Pathology, Akron Children’s Hospital, Akron, OH USA; 3 Northeast Ohio Medical University, Rootstown, OH USA; 4 Department of Nephrology, Weill Cornell Medicine/New York Presbyterian, New York, USA

**Keywords:** Hemolytic uremic syndrome, Gemcitabine, Thrombotic microangiopathy, Renal failure, Pancreatic cancer

## Abstract

**Background:**

Thrombotic microangiopathy (TMA) secondary to gemcitabine therapy (GiTMA) is a very rare pathology that carries a poor prognosis, with nearly half of the cases progressing to end stage renal disease. GiTMA is most commonly associated with adenocarcinomas, most notably pancreatic cancers. The mainstay of management is withdrawal of the offending drug and supportive care. Plasmapheresis has a limited role and hemodialysis may help in the management of fluid overload secondary to renal failure. Furthermore, a C5 inhibitor, eculizumab, has been successfully used in the treatment of GiTMA.

**Case presentation:**

A 64-year-old Caucasian female with history of pancreatic adenocarcinoma on gemcitabine chemotherapy presented with signs and symptoms of fluid overload and was found to have abnormal kidney function. Her BP was 195/110 mmHg, serum creatinine 4.48 mg/dl, hemoglobin 8.2 g/dl, platelets 53 × 10^3^/cmm, lactate dehydrogenase 540 IU/L, and was found to have schistocytes on blood film. A diagnosis of TMA secondary to gemcitabine therapy was suspected. Hemodialysis for volume overload and daily plasmapheresis were initiated. After six days of plasmapheresis, renal function did not improve. Further work up revealed ADAMTS 13 activity >15%, low C3, and stool culture and Shiga-toxin PCR were negative. Renal biopsy was consistent with TMA. Gemcitabine was discontinued, but renal function failed to improve and eculizumab therapy was considered due to suspicion of aHUS. Serum creatinine >2.26 mg/dl and a platelet count of >/= 30 × 10^9^/L is highly suggestive of aHUS, while TTP is more likely when creatinine is <2.26 mg/dl and platelet count of <30 × 10^9^/L. She received intravenous eculizumab for eight months, which resulted in significant improvement of renal function. Other markers of hemolysis, namely LDH and bilirubin, also rapidly improved following eculizumab therapy. Plasmapheresis and hemodialysis were discontinued after two and eight weeks of initiation respectively.

**Conclusion:**

Chemotherapy induced TMA is very rare and requires a high index of clinical suspicion for timely diagnosis. Discontinuation of the offending drug and supportive care is the main stay of treatment; however, eculizumab has been shown to be beneficial in GiTMA. Further research is required to validate this approach.

## Background

Thrombotic microangiopathies (TMA) are group of disorders recognized by microangiopathic hemolytic anemia (MAHA), renal failure, and thrombocytopenia. Some of the diseases that fall under the umbrella of TMA, which was first described in 1924, are thrombotic thrombocytopenic purpura (TTP), hemolytic uremic syndrome (HUS), disseminated intravascular coagulation (DIC), and malignant hypertension [1, 2]. Though the clinical and laboratory picture often overlaps, the underlying etiology differentiates TTP and HUS. HUS is most likely if initial presentation is severe renal failure. In TTP, lack of metalloprotease ADAMTS13, which normally cleaves large multimers of von Willebrand factor into smaller subunits on the endothelial cell surface, produces thrombi in microvasculature of most organs including the central nervous system. HUS results from endothelial damage due to bacterial toxins, particularly Shiga toxin produced by *E. coli* O157:H7 and Shigella dysenteriae infections. This results in microvascular thrombi with predilection to renal vasculature. Histopathology of thrombi in HUS and DIC shows fibrin and platelets, whereas thombi are composed of predominantly platelets with little or no fibrin in TTP [2].

HUS is a rare disorder first described in 1955 and is characterized by the triad of hemolytic anemia, thrombocytopenia, and acute kidney injury [2]. HUS has high mortality rates of 10–40% and in some cases up to 60–70% [3]. HUS is divided into two types; diarrhea positive or typical HUS and diarrhea negative or atypical HUS (aHUS) [4, 5]. Atypical HUS (aHUS) is a rare variant of TMA that carries a poor prognosis with nearly half of the cases progressing to end stage renal disease necessitating renal replacement therapy [6]. The primary organ affected is the kidney; however, 20% of patients have extrarenal expression of aHUS, with the central nervous system being most common, followed by cardiovascular, pulmonary, gastrointestinal, skin and skeletal muscles involvement [7]. The underlying pathology in aHUS is mutation of complement regulatory genes resulting in uncontrolled complement activation and formation of microvascular thrombi in a majority of cases [6].

Certain conditions such as infection (streptococcus pneumonia, HIV), connective tissue disease, pregnancy, malignancy, and drugs (bleomycin, cisplatin, gemcitabine, mitomycin C, tacrolimus, cyclosporine, anti-VEGF agents, interferon, etc.) may also predispose to TMA [6]. The primary modality of treatment is discontinuation of causative agent and supportive care. Here we report a case of eculizumab use for gemcitabine induced TMA in a pancreatic cancer patient who failed to show renal function recovery with standard treatment.

## Case presentation

A 64-year-old Caucasian female, who initially presented with recurrent abdominal pain, was found to have pancreatic adenocarcinoma on histopathology following distal pancreatectomy. The patient was subsequently considered for chemotherapy by the oncologist and started on intravenous gemcitabine with a dose of 1000 mg/m^2^/week for three weeks a month. Patient completed three cycles of gemcitabine therapy and kidney function was found to be abnormal during the fourth cycle with signs and symptoms of volume overload. Her blood pressure was 195/110 mmHg, serum creatinine was 4.48 mg/dl (normal 0.6–1.5 mg/dl) and blood urea nitrogen (BUN) 48 mg/dl (normal 7–25 mg/dl). Hematological work up showed a drop in hemoglobin (Hb) level from baseline and thrombocytopenia. Hb level was 8.2 g/dl (normal 14.4–16.6 g/dl), and platelets count decreased to 53 × 10^3^/μL (182–369 × 10^3^/μL). Lactate dehydrogenase (LDH) levels were elevated at 540 IU/L (normal 110–240 IU/L), total bilirubin 1.1 mg/dl (normal 0.3–1 mg/dl), haptoglobin level dropped to 27 mg/dl (normal 41–165 mg/dl) and blood film showed presence of schistocytes suggestive of MAHA.

**Fig. 1:**
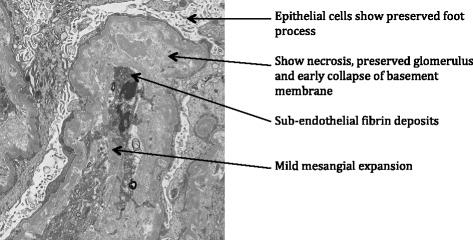
Renal biopsy showing preserved foot process and glomerulus with necrosis and early collapse of basement membrane, sub-endothelial fibrin deposits, mild mesangial expansion

**Fig. 2:**
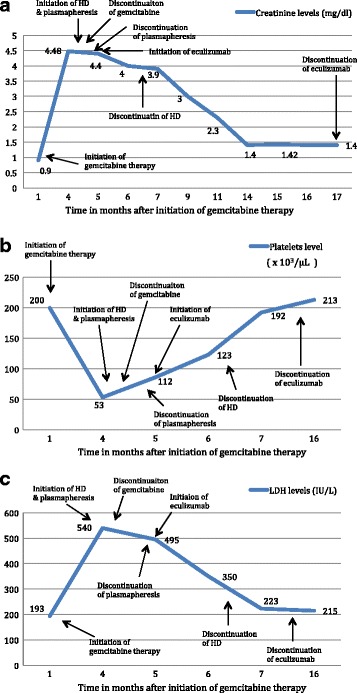
**a** Demonstration of serum creatinine levels in relation to time since initiation of gemcitabine therapy and different interventions. **b** Demonstration of platelets level in relation to time since initiation of gemcitabine therapy and different interventions. **c** Demonstration of LDH level in relation to time since initiation of gemcitabine therapy and different interventions. HD – hemodialysis

A diagnosis of TMA secondary to gemcitabine therapy was suspected due to the timing of presentation of TMA clinical features after initiation of chemotherapy. Patient was started on daily plasmapheresis with fresh frozen plasma (1.5 times the total plasma volume) and hemodialysis initiated for volume overload. Further work up included serum ADAMTS 13 activity, stool culture, Shiga-toxin PCR, C3 levels, and renal biopsy. After six days of plasmapheresis, hematological parameters improved with platelet count of 102 × 10^3^/μL but renal functions did not improve. ADAMTS 13 activity was >15%, C3 level was low, stool culture and Shiga-toxin PCR were negative. Patient had renal biopsy as platelet count was above 100,000/μL, which avoids the risk of bleeding and hematoma formation. Renal biopsy (Fig. 1) showed sub-endothelial fibrin deposits, mild mesangial expansion, and early collapse of the basement membrane. Immunofluorescence showed no glomerular staining to indicate deposition of IgG, IgA, IgM or complement components C3 or C_1_q. Blood vessels showed staining for C3 and increased glomerular fibrin deposits. A diagnosis of TMA secondary to gemcitabine therapy was confirmed based on the clinical and laboratory evidence, and further genetic testing for complement mutations was not pursued. A decision was made to stop gemcitabine; however, renal failure did not improve for four weeks following discontinuation. After discussing with the oncologist, eculizumab therapy was considered due to features suggestive of aHUS (absence of Shiga-toxin, ADAMTS13 activity >15% and renal biopsy showing C3 staining in blood vessels and increased glomerular fibrin deposits) and its previous successful use in the setting of drug-induced-TMA as evidenced in published reports [8]. Patient was given meningococcal vaccine and started on meningococcal prophylaxis with ciprofloxacin 250 mg oral daily. She received one dose of intravenous eculizumab 900 mg every week for four weeks and 1200 mg at week 5 followed by 1200 mg every two weeks for a total of eight months. The patient’s renal function showed significant improvement allowing discontinuation of plasmapheresis and hemodialysis after two and eight weeks of initiation respectively. Creatinine clearance improved from 14 to 40 ml/min/1.7 m^2^ and serum creatinine was 1.4 mg/dl by the last dose of eculizumab. Other markers of hemolysis, namely LDH and bilirubin, improved more rapidly after initiating eculizimab. At that time, the patient was taken off eculizumab and further follow up with her oncologist was recommended. Trends in lab parameters in relation to the timing of intervention are shown in Fig. 2. Eight months after discontinuing eculizumab, patient died due to cancer-related complications.

## Discussion

Casper et al. reported the first case of gemcitabine related TMA/HUS in a 55-year-old man with pancreatic adenocarcinoma after receiving gemcitabine treatment for a year [9]. Gemcitabine, a deoxycytidine analog antimetabolite, is a commonly used chemotherapeutic agent for cancers of the pancreas, breast, lung and ovaries [3]. Intracellular phosphorylation by deoxycytidine kinase activates gemcitabine and inhibits DNA polymerase, thereby inhibiting DNA synthesis, resulting in apoptosis of cells [10, 11]. There are no structural similarities between gemcitabine and other chemotherapeutic drugs that have been implicated in the pathogenesis of TMA, such as cisplatin, bleomycin, or liposomal doxorubicin, indicating that chemotherapy induced TMA is not restricted to any one particular drug class [3].

Incidence of gemcitabine induced TMA (GiTMA) varies from 0.008% to 0.078% with an overall incidence of 0.015%, though GiTMA may be grossly underreported [3]. In a study by Muller et al., the incidence of GiTMA was reported to be 1.4% [12], and in a systematic review by Izzedine et al., the incidence of GiTMA was noted to be 0.4% [13]. GiTMA has been noted to be more common in males aged 50–69 years, but this can be a reflection of the natural course of the malignancy for which gemcitabine was implicated [14].

The pathogenesis of GiTMA is still unclear and very speculative [10, 15]. One of the mechanisms might be direct damage to the endothelium by the drug with consequent activation of the clotting system. This can be explained by higher levels of nitrate found in chemotherapy induced TMA, which correlates with endothelial injury [16]. The injured endothelium release large amounts of von Willebrand factor (vWF) multimers which lead to platelet aggregation and fibrin deposition [15]. Renal and cerebral vessels are involved more in comparison to the hepatic and pulmonary microvasculature due to the expression of CD36 (thrombospondin receptor) on the endothelium of renal and cerebral microvasculature [13]. ADAMTS13 is a vWF cleaving metalloprotease that has been found to be severely deficient in patients with TTP [11]. On the contrary, ADAMTS13 levels are found to be low in patients with metastatic cancer and GiTMA, suggesting that gemcitabine induces the formation of antibody against ADAMTS13 enzyme [13]. Damaged endothelium along with the reduction in ADAMTS13 activity lead to elevated levels of vWF multimers resulting in platelet and fibrin thrombi. The occlusion of microvasculature by platelets and fibrin thrombi leads to erythrocyte fragmentation and renal failure [14]. Another theory could be that circulating immune complexes containing the tumor antigen deposit at various sites and trigger the local intravascular coagulation system [15].

The clinical presentation of chemotherapy induced TMA is highly variable depending upon the inciting drug and generally presents 4 to 8 weeks following chemotherapy initiation [15]; however, studies have reported its occurrence up to 10 months after the initiation of gemcitabine therapy [17]. There is no fixed dose relationship but most studies have demonstrated that a cumulative dose in excess of 20,000 mg/m2 to be associated with the development of GiTMA. Hypertension, advanced stage of cancer, and prolonged gemcitabine treatment have been noted to be predisposing factors for the development of GiTMA [18].

Two types of clinical presentations of TMA related to chemotherapy and malignancy have been described. One is a more acute form with rapid renal failure, dramatic hemolysis, and severe thrombocytopenia. The second form is more indolent and presents with slowly progressive uremia and mild thrombocytopenia [19]. GiTMA often presents with dyspnea secondary to non-cardiogenic pulmonary edema, new onset hypertension, or exacerbation of underlying hypertension [12, 15, 20, 21]. In the analyses by Walter et al., hypertension and new-onset dyspnea were present in >50% cases of GiTMA. Renal toxicity is also a cardinal feature of TMA with proteinuria and hematuria being the most common manifestations. Livedo reticularis can also be seen in GiTMA due to endothelial damage and intravascular fibrin deposition leading to local ischemia [22].

Laboratory findings suggestive of MAHA include elevation in creatinine and LDH, proteinuria, microscopic hematuria, decreased haptoglobin and the presence of reticulocytes [10, 13, 15]. Peripheral smears show the presence of fragmented erythrocytes (schistocytes), burr cells as well as microspherocytes [15]. The diagnosis of chemotherapy induced TMA can be challenging due to myelosupression from chemotherapy itself that can present with similar findings of anemia and thrombocytopenia. However, signs of hemolysis (schistocytes, burr cells, elevated reticulocytes and LDH) and negative Coombs test aids the diagnosis of TMA [20]. Reticulocyte count may be low because of prior transfusions or myelosupression [17]. Furthermore, studies have demonstrated that serum creatinine >2.26 mg/dl and a platelet count of >/= 30 × 10^9^/L is highly suggestive of aHUS, and TTP is more likely when creatinine is <2.26 mg/dl and platelet count of <30 × 10^9^/L. Since aHUS carries higher rates of morbidity and mortality, this approach may help physicians to diagnose and initiate treatment early while awaiting results of ADAMTS13 activity and when Shiga-toxin is absent [23]. Additionally, kidney biopsy in MAHA demonstrates thickening of the glomerular capillary wall along with fibrin deposition in the capillaries and arterioles, giving the characteristic tram-track appearance [14, 15, 22]. Immunofluorescence demonstrates the deposition of IgM and C3 in the mesangium, and electron microscopic examination shows pronounced endothelial injury [22]. Renal biopsy is not necessary for the diagnosis of TMA but is preferred by many histologists as it may aid in the quantification of irreversible sclerotic lesions that may help in prognostication of patients with GiTMA [13, 24]. Because these chemotherapeutic drugs are often used for treatment of various malignancies, differentiating malignancy related and chemotherapy associated TMA can be difficult to ascertain. Experimental studies have shown, however, that markers such as tumor necrosis factor alpha, interleukin-1 beta, interleukin 6, vWF antigen and low molecular weight vWF multimers may help differentiate chemotherapy related TMA from malignancy related TMA [20].

The main stay of management in chemotherapy induced TMA is discontinuation of the offending drug and treatment of renal failure with dialysis if necessary [24]. Role of plasmapheresis in the management of chemotherapy induced TMA is limited, and 50% of patients still progress to end stage renal disease (ESRD) [5]. Protein A immunoadsorption is another modality that has demonstrated improvement in chemotherapy induced TMA [12]. Rituximab has also been used for the treatment of GiTMA; the exact mechanism by which it is beneficial is unclear, but it most likely decreases immune complexes by targeting B-lymphocytes [25, 26].

Eculizumab is a novel C5 inhibitor used for aHUS due to complement factor abnormalities as well as PNH [8, 27]. It prevents the formation of C5a and membrane attack complex (C5b-C9) in the alternate pathway of the complement system, thereby inhibiting inappropriate systemic coagulation [8]. Eculizumab has also been tried in the management of GiTMA with significant improvement in renal function and resolution of hematological parameters [8]. Our patient demonstrated significant improvement in renal function and hematological parameters after initiation of eculizumab therapy allowing for discontinuation of hemodialysis and plasmapheresis. Although therapeutic plasma exchange (TPE) has not been shown to be effective for gemcitabine-associated TMA [13], it is important to note that it may have helped recovery in our patient. However, it is less likely that there is an antibody-mediated cause in GiTMA [28], supporting the thought that recovery was most likely in response to eculizumab than TPE. Several case reports have demonstrated recovery of renal function in GiTMA after use of eculizumab. Rogier et al. describes renal function recovery and return of hematologic parameters to baseline after seven doses of eculizumab in a patient with GiTMA that failed to respond to plasma exchange [29]. Turner et al. reports two patients with GiTMA that were successfully treated with eculizumab. One of these two patients was able to restart gemcitabine treatment without recurrence of TMA [30]. Lopez et al. presents two patients with gemcitabine- induced hemolytic anemia, one of whom was treated with eculizumab and the other patient was treated with hemodialysis and plasmapheresis. The patient treated with eculizumab showed renal and hematologic recovery and was able to stop hemodialysis after 7 doses of treatment. The patient treated with conventional treatment remained on dialysis for two months and progressed to stage IV chronic kidney disease [31]. Further large-scale research is needed to study the efficacy of eculizumab in the management of GiTMA.

Eculizumab is a notably costly drug, priced around $18,000 per dose. The use of eculizumab must be judicious in context of overall patient prognosis. Genetic testing for complement regulatory mutations may also be done in subgroups of patients suffering from gemcitabine-associated TMA that would allow for more targeted therapy with eculizimab.

## Conclusion

GiTMA is a very rare and highly fatal condition with mortality rates ranging from 50 to 70%. Physicians should have a high index of suspicion to diagnose GiTMA early in the course of the disease. Mainstay of management is discontinuation of gemcitabine therapy and supportive care. Eculizumab is a C5 inhibitor, which has been tried in GiTMA with improvement in laboratory parameters. However, further research is required to validate this treatment strategy.
